# Gene model for the ortholog of *yellow-f2* in *Drosophila dunni*

**DOI:** 10.17912/micropub.biology.002348

**Published:** 2026-09-15

**Authors:** Hope Freshwater, Pablo Chialvo, Clare Scott Chialvo

**Affiliations:** 1 Biology, Appalachian State University, Boone, North Carolina USA

## Abstract

We developed a gene model for the *yellow-f2* ortholog in the ASM1815212v1 Genome Assembly (GenBank Accession: GCA_018152125.1 ) of *Drosophila dunni* . This ortholog was characterized as part of a developing dataset for a comparative study of detoxification gene family evolution in the *immigrans* - *tripunctata* radiation of the genus *Drosophila* using an adapted Genomics Education Partnership gene annotation protocol for Course-based Undergraduate Research Experiences.

**Figure 1. Genomic neighborhood and gene model for yellow-f2 ortholog in D. dunni:** (A) **Synteny comparison of the genomic neighborhoods for *yellow-f2* in *Drosophila melanogaster* and *Drosophila dunni* .** Thin underlying arrows indicate which DNA strand the target gene – *yellow-f2* – is located on in *D. melanogaster* (top) and *D. dunni* (bottom). The thin arrow pointing to the left indicates that *yellow-f2* is on the negative strand in both *D. melanogaster* and *D. dunni* . The wide gene arrows pointing in the same direction as *yellow-f2* are on the same strand relative to the thin underlying arrows, while wide gene arrows pointing in the opposite direction of *yellow-f2* are on the opposite strand relative to the thin underlying arrows. White gene arrows in *D. dunni* indicate orthology to the corresponding gene in *D. melanogaster* , while black gene arrows indicate non-orthology, and gray arrows indicate that the gene is present in both genomic neighborhoods but not syntenic. Gene symbols given in the *D. dunni* gene arrows indicate the orthologous gene in *D. melanogaster* , while the locus identifiers are specific to *D. dunni* . (B) **Gene Model in GEP UCSC Track Data Hub** (Raney et al., 2014). The coding-regions of *yellow-f2* in *D. dunni* . are displayed in the User Supplied Track (red); coding sequences (CDS) are depicted by thick rectangles and introns by thin lines with arrows indicating the direction of transcription. Subsequent evidence tracks include Spaln of *D. melanogaster* Proteins (purple, alignment of Ref-Seq proteins from *D. melanogaster* ), Coding Regions Predicted by Augustus (dark blue), GeMoMa (teal), and NSCAN PASA-EST (dark green), and RNA-Seq from mixed sex adult flies (brown; alignment of Illumina RNA-Seq reads from *D. dunni* – Erlenbach et al., 2023). (C) **Dot Plot of yellow-f2-PA in *D. melanogaster* ( *x* -axis) vs. the orthologous peptide in *D. dunni* ( *y* -axis).** Amino acid number is indicated along the left and bottom; CDS number is indicated along the top and right, and CDSs are also highlighted with alternating colors. Line breaks in the dot plot indicate areas of with low sequence identity between species.

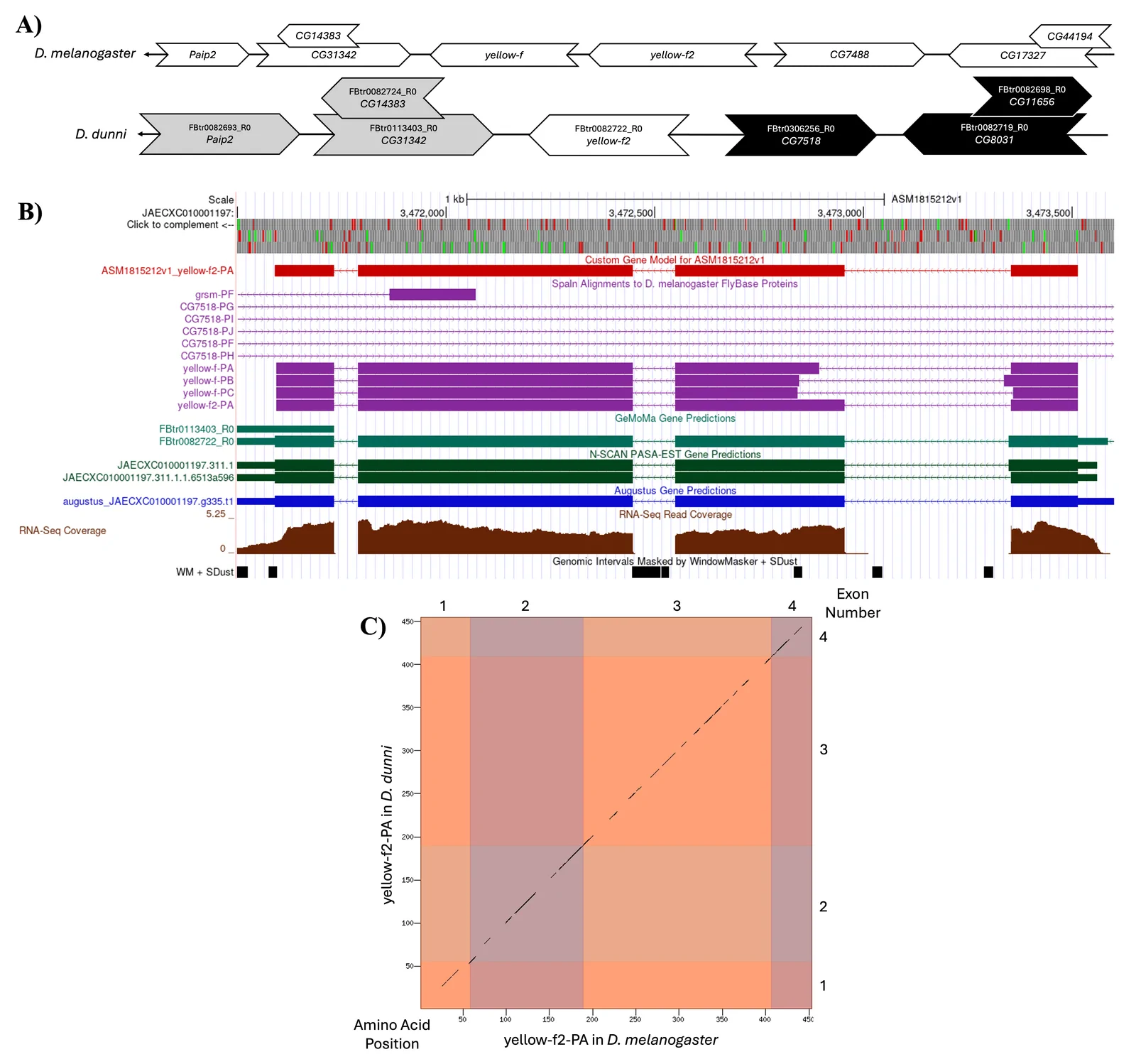



## Description

***Introduction***

**Table:** 

*This article reports a predicted gene model generated by undergraduate work using a structured gene model annotation protocol defined by the Genomics Education Partnership (GEP; thegep.org) for Course-based Undergraduate Research Experience (CURE). The following information in quotes may be repeated in other articles submitted by participants using the same GEP CURE protocol for annotating Drosophila species orthologs of Drosophila melanogaster detoxification genes.* ** ** “Within insects, detoxifying xenobiotics and host secondary metabolites is a three-phase process that involves functionalization, conjugation, and excretion of these compounds. Expansions of known detoxification gene families ( *e.g.* , cytochrome P450s) are associated with diet breadth and insecticide resistance (Ranson et al., 2002; Després et al., 2007; Rane et al., 2016). With the increasing availability of high-quality genomes for non-model organisms, including *Drosophila* species beyond *D. melanogaster* , it is now possible to perform large scale comparative studies (Robinson et al., 2011; Kim et al., 2021; Threfall and Baxter, 2021). Careful manual annotation and curation of gene models can improve upon computational gene predictions in non-model species, which aids the accuracy of studies on gene and genome evolution (Mudge and Harrow, 2016; Tello-Ruiz et al., 2019). To aid in these annotations, the Genomics Education Partnership (thegep.org) developed a curriculum involving web-based tools that allow undergraduates to engage in authentic course-based research focused on manually annotating genes in non-model species (Rele et al., 2023). The orthologous gene models, including the one presented here, then provide a reliable basis for further evolutionary genomic analyses when made available to the scientific community. The gene ortholog described here in *D. dunni* for *yellow-f2* , a member of the intramolecular oxidoreductase gene family was characterized as part of a developing dataset for a comparative study of detoxification gene families in the *immigrans* - *tripunctata* radiation of the genus *Drosophila* .” (Williams et al., 2026) “Within the subgenus *Drosophila* , *D. dunni* Townsend and Wheeler 1955 is placed in the *dunni* subgroup of the *cardini* species group in the *immigrans-tripunctata* radiation (Heed and Krishnamurthy, 1959; Bächli, 2005). Species in the *dunni* subgroup, including *D. dunni* , are distributed across the Caribbean (Heed and Krishnamurthy, 1959). Members of the *cardini* group primarily feed and develop on fruit and flowers (Markow and O'Grady, 2008). While some mushroom-feeding *cardini* subgroup members tolerate the mushroom toxin α-amanitin (Stump et al., 2011), the fruit/flower feeding *dunni* subgroup species do not (Erlenbach et al., 2023).” (Williams et al., 2026) Intramolecular oxidoreductases are isomerases that oxidize one part of a substrate while concurrently reducing another part of the same molecule, producing no net change in overall oxidation state. The family includes genes involved in dopachrome/dopaminechrome tautomerase activity. This process converts reactive cyclized catecholamine-quinone intermediates into dihydroxyindoles that feed insect eumelanin synthesis, a biosynthetic step that simultaneously channels cytotoxic quinones out of the cell during cuticular melanization (Han et al., 2002; Barek et al., 2022). *yellow-f2* is a member of the intramolecular oxidoreductase gene family that is active in the cuticular eumelanin pathway downstream of tyrosine hydroxylase and dopamine decarboxylase. Transcripts are present at all stages but peak in later pupae and adults (Han et al., 2002).

We propose a gene model for the *D. dunni* ortholog of the *D. melanogaster* *yellow-f2* gene. The genomic region of the ortholog corresponds to the FBtr0082722_R0 GeMoMa prediction in the ASM1815212v1 Genome Assembly of *D. dunni* ( GCA_018152125.1 – Kim et al., 2021). This model is based on mixed sex, adult RNA-Seq data from *D. dunni* (Erlenbach et al., 2023; https://doi.org/10.5061/dryad.hdr7sqvq2) and *yellow-f2* in *D. melanogaster* using FlyBase release FB2024_02 ( GCA_000001215.4 ; Öztürk-Çolak et al., 2024).

***Synteny***

The reference gene, *yellow-f2 ,* occurs on chromosome 3R in *D. melanogaster* and is flanked upstream by *CG7488 , CG17327* , and *CG44194* (nested within *CG17327* ) and downstream by *CG31342* , *CG14383* (nested within *CG31342* ), and *polyA-binding protein interacting protein 2* ( *Paip2* ). The *tblastn* search of *D. melanogaster* *yellow-f2* (query) against the *D. dunni* genome assembly (GenBank Accession: GCA_018152125.1 ; subject) placed the putative ortholog of *yellow-f2* within contig_1225 (JAECXC10001197.1) which corresponds to GeMoMa gene prediction FBtr0082722_R0 (E-value: 0; percent identity: 71.08% as determined by *blastp* ). The putative ortholog is flanked upstream by the GeMoMa predictions FBtr0306256_R0, FBtr0082719_R0, and FBtr0082698_R0 (nested within FBtr0082719_R0), which correspond to *CG7518* , *CG8031 ,* and *CG11656* in *D. melanogaster* (E-value: 0, 0, and 2e-97; identity: 65.07%, 89.80%, and 51.39%, respectively, as determined by *blastp* ; Figure 1A ; Altschul et al., 1990). The putative ortholog of *yellow-f2* is flanked downstream by the GeMoMa FBtr0113403_R0, FBtr0082724_R0 (nested within FBtr0113403_R0), and FBtr0082693_R0 which correspond to *CG31342* , *CG14383* , and *Paip2* in *D. melanogaster* (E-value: 0, 4e-55, 1e-78; identity: 66.51%, 33.24%, 91.13% respectively, as determined by *blastp* ). The putative ortholog assignment for *yellow-f2* in *D. dunni* is supported by the following evidence: The *tblastn* results are of good quality, and all CDSs and isoforms found in *D. melanogaster* also appear to be present in *D. dunni.* The gene predictions surrounding the *yellow-f2* ortholog are partially conserved. The three downstream genes are shifted by one position (e.g, *CG31342* is the first downstream gene compared to second downstream in *D. melanogaster* ). Likewise, the three genes upstream of the putative ortholog are shifted by two positions (e.g., *CG7518* is one upstream from the target, whereas it is three upstream in *D. melanogaster* ). As such, we conclude that FBtr0082722_R0 is an ortholog of *yellow-f2* in *D. dunni* ( Figure 1A ).

***Protein Model***

*yellow-f2* in *D. dunni* has four coding sequences (CDS) within the genomic sequence. The only unique protein sequence yellow-f2-PA is translated from one messenger RNA ( Figure 1B ). Relative to the ortholog in *D. melanogaster* , the CDS number and protein isoform count are conserved *.* The sequence of yellow-f2-PA in *D. dunni* has 70.6% identity (79.5% similarity) with the protein-coding isoform yellow-f2-PA in *D. melanogaster* , as determined by *blastp* ( Figure 1C ). This level of divergence is not surprising given that *D. dunni* and *D. melanogaster* belong to two separate subgenera ( *Drosophila* and *Sophophora* respectively) that diverged approximately 45-60 MYA (Russo et al., 1995; Tamura et al., 2004; Obbard et al., 2012). Coordinates of this curated gene model are archived in the CaltechDATA repository (see “Extended Data” section below).

## Methods

The annotation methods used in this project are adapted from those described in Rele et al. (2023), which includes algorithms, database versions, and citations for the complete annotation process developed for the Pathways Project. The methods for the current project are detailed in brief below with notes on significant differences between this protocol and the one described in Rele et al. (2023). The students use the GEP instance of the UCSC Genome Browser v.435 (https://gander.wustl.edu; Kent et al., 2002; Raney et al., 2024) to examine the genomic neighborhood of their reference detoxification gene in the *D. melanogaster* genome assembly (Aug. 2014; BDGP Release 6 + ISO1 MT/dm6). Students obtain the protein sequence for the *D. melanogaster* target gene for a given isoform and use a *tblastn* search of the sequence against their target *Drosophila* species genome assembly ( *D. dunni* ( GCA_018152125.1 – Kim et al., 2021)) on the NCBI BLAST server (https://blast.ncbi.nlm.nih.gov/Blast.cgi, Altschul et al., 1990) to identify the putative ortholog location. Students compare the genomic neighborhood of the putative ortholog to that of the reference gene in *D. melanogaster* . This local synteny analysis includes a minimum of two upstream and two downstream genes relative to the potential ortholog. As no RefSeq protein data is available for these species, comparisons are based on gene predictions that correlate with gene expression data in the putative ortholog neighborhood. Using the multiple alignment tracks feature in the Genome Browser, students examine other sets of genomic evidence, including Spaln alignment of *D. melanogaster* proteins, multiple gene prediction tracks (e.g., GeMoMa, Augustus, NSCAN PASA-EST), and mixed sex RNA-Seq adult expression data from the target species generated by Erlenbach et al. (2023; https://doi.org/10.5061/dryad.hdr7sqvq2). Information on the genomic structure information (e.g., CDSs, intron-exon number, number of isoforms) for the reference gene in *D. melanogaster* is retrieved using Gene Record Finder (https://gander.wustl.edu/~wilson/dmelgenerecord/index.html; Rele et al *.,* 2023). To determine approximate splice sites within the target gene, a *tblastn* search using the CDSs from the *D. melanogaste* r reference gene against the putative ortholog location (10kb up- and downstream of the target gene prediction). Coordinates of the CDS(s) are refined by examining aligned RNA-Seq data, identifying canonical splice site sequences, and ensuring the maintenance of an open reading frame. Students confirm the biological validity of their target gene model using the FlySeq Gene Model Checker (https://gander2.wustl.edu/~wilson/genechecker-flyseq/), which compares the hypothesized target gene model's structure and translated sequence against the *D. melanogaster* reference gene. At least two independent models for this gene are generated. These models are reconciled by a third independent researcher to produce the final model presented here. Note: comparison of 5' and 3' UTR sequence information is not included in this GEP CURE protocol.

## Data Availability

Description: Zipped archive containing FASTA, PEP, and GFF files for yellow-f2 model in D. dunni. Resource Type: Model. DOI: https://doi.org/10.22002/y3z7h-z8t72
